# Calcium application synergistically enhances yield and nutritional quality in waxy maize

**DOI:** 10.3389/fpls.2026.1777765

**Published:** 2026-03-11

**Authors:** Aiju Meng, Zhengwei Yang, Pengao Yu, Rui Guo, Jingyan Liu, Wei Jiang, Jiaxin Shi, Jin Du, Chunyang Xiang

**Affiliations:** 1 Tianjin Key Laboratory of Intelligent Breeding of Major Crops, College of Agronomy Resources and Environment, Tianjin Agriculture University, Tianjin, China; 2 Maize Research Institute, Ordos Academy of Agricultural and Animal Husbandry Sciences, Ordos, China; 3 Tianjin Nan'ao Seed Co., Ltd., Tianjin, China

**Keywords:** calcium chloride, nutritional quality, photosynthetic characteristics, waxy maize, yield

## Abstract

**Introduction:**

Optimizing the yield and nutritional quality of waxy maize is essential to meet evolving consumer demands and ensure food security. Calcium plays a vital role in plant physiology, but its foliar application effects on the synergistic improvement of yield and kernel nutritional traits across diverse waxy maize hybrids remain underexplored.

**Methods:**

This study investigated the effects of foliar-applied calcium chloride (CaCl_₂_, 18 mmol/L, applied at the V12 stage) on 36 hybrid varieties of waxy maize. Agronomic traits, photosynthetic parameters, and grain nutritional components were measured and analyzed.

**Results:**

The treatment slightly suppressed plant and ear height, but significantly increased ear length, ear diameter, and fresh weight per ear. Photosynthetic analysis revealed that 34 out of 36 hybrids exhibited a significant increase in net photosynthetic rate (ranging from 4.5% to 230%), while two hybrids showed non-significant increases. SPAD values showed non-significant increases, suggesting that calcium enhanced the operational efficiency of the photosynthetic apparatus rather than chlorophyll content. Concurrently, the treatment significantly enhanced kernel nutritional quality. Soluble sugar content increased by 1.1–17%, crude protein by 1.5–24.8%, and key micronutrients—zinc (2.7–13.8%), iron (1.7–15.4%), and calcium (1.6–15.3%)—were significantly elevated. Correlation analysis revealed that soluble sugar content was highly significantly and positively correlated with ear length and diameter, and that increases in soluble sugar and protein were significantly associated with micronutrient accumulation.

**Discussion:**

These findings indicate that exogenous CaCl₂ optimizes source–sink balance by improving photosynthetic efficiency and assimilate partitioning, thereby driving a synergistic enhancement of yield and nutritional quality in waxy maize. This work provides a physiological basis and a practical agronomic strategy for the targeted biofortification and yield improvement of maize.

## 1 Introduction

Waxy maize (*Zea mays* L. *sinensis Kulesh*) is highly preferred by consumers for its unique amylopectin composition and soft, sticky texture (Li et al., 2022; Luo et al., 2024). Research has demonstrated that waxy maize kernels contain significantly higher levels of vitamins, proteins, lysine, and mineral elements (such as iron and zinc) compared to conventional maize (Baveja et al., 2021; Dang et al., 2023). The micronutrient profile fundamentally governs the nutritional value and commercial viability of waxy maize. The core demand of the waxy maize industry has shifted from solely pursuing yield to integrating high yield, superior quality, and enhanced nutritional functionality. However, the persistent yield-quality trade-off in conventional breeding and production remains a key bottleneck constraining its overall value enhancement. Therefore, exploring novel agronomic approaches that can coordinately regulate yield formation and nutrient accumulation is of crucial significance for meeting consumer demands and driving industry development.

Calcium ions (Ca²^+^) function as essential secondary messengers that play critical roles in regulating plant growth and stress responses (Wang et al., 2023; Gai et al., 2024). While its canonical roles in cellular structure maintenance and enzymatic regulation are well established (Sharma and Komatsu, 2002; Yan et al., 2025), emerging research reveals Ca²^+^-mediated signaling networks fundamentally govern source-to-sink carbon partitioning, endosperm development, and mineral acquisition pathways (Frattini et al., 1999; Manimaran et al., 2015; Arif et al., 2016; Weng et al., 2022). While exogenous Ca²^+^ application is extensively documented for enhancing postharvest attributes in horticultural crops (Hou et al., 2021; Yan et al., 2025). Its efficacy in improving kernel quality parameters in cereals—particularly waxy maize—is yet to be systematically investigated. Existing studies predominantly address Ca²^+^’s protective roles under abiotic stresses (Cao et al., 2020; Li et al., 2023), with limited insights into its regulatory interplay with yield-determining traits (ear architecture) and nutrient partitioning kinetics during kernel filling. A critical knowledge gap persists regarding the phenological specificity of Ca²^+^ foliar sprays, particularly whether the V12 stage, a developmental phase concurrently governing ear formation and assimilate remobilization, constitutes an optimal intervention window for concurrent yield and quality enhancement.

Calcium chloride (CaCl_2_) is widely used as a foliar calcium fertilizer due to its high solubility, low cost, and excellent bioavailability. When applied via foliar spraying, calcium ions can penetrate leaf tissues directly through stomata and the cuticle, thereby entering the plant’s metabolic cycle, effectively avoiding soil compaction issues associated with soil-based calcium application. The bell stage is a critical phase for ear floret differentiation and kernel sink capacity formation in maize. Calcium may regulate kernel development by altering endogenous cytokinin distribution, thereby affecting kernel number and size. Its involvement in carbohydrate partitioning and protein biosynthesis (Cheng et al., 2002; Mukherjee et al., 2017; Zhu et al., 2024), suggests that foliar calcium application could concurrently improve both mineral (Ca, Fe, Zn) and organic (protein, sugars) nutrient accumulation in kernels.

In this study, we investigated the effects of foliar application of 18 mmol/L CaCl_2_ on 36 waxy maize hybrids, analyzing key agronomic traits and nutritional quality parameters. The research addresses two fundamental questions: First, how does CaCl_2_ treatment enhance yield potential by coordinating ear morphological development? Second, can this treatment simultaneously improve both micronutrient content (hidden nutrition, e.g., Fe, Zn) and macronutrient quality (visible traits, e.g., proteins, sugars) in kernels, thereby achieving synergistic improvement of yield and nutritional value?”

## 2 Materials and methods

### 2.1 Plant materials and cultivation

36 waxy maize hybrids were provided by Tianjin Agricultural University. Calcium chloride (CaCl_2_, purity > 98%) was purchased from Solarbio Science & Technology Co., Ltd. (Beijing, China).

Field experiments were conducted in Tianjin (116.97° E, 39.09° N). The region experiences a temperate continental monsoon climate, with an annual average temperature of 11.6 °C, annual precipitation exceeding 500 mm, and a frost-free period of 190 days. The experimental field soil was classified as a Fluvo-aquic soil, with a pH of 8.00 and an electrical conductivity of 376.00 µS/cm. The soil contained 42.62 g/kg of organic matter, 1.82 g/kg of total nitrogen, 2.21 g/kg of total phosphorus, and 21.32 g/kg of total potassium. The initial soil available calcium (Ca) content was 9.56 g/kg.

### 2.2 Experimental design and treatments

The trial was sown on May 2, 2022, employing a randomized complete block design with three replicates. Each replicate comprised 36 plots corresponding to the 36 waxy maize hybrids. Plots were arranged as three-row sections (row length: 5 m, row spacing: 0.6 m) with 17 hills per row. The two treatments consisted of a Control (foliar spray with deionized water) and a Calcium Chloride treatment (18 mmol/L CaCl_2_ applied at the V12 stage), with each replicate plot receiving a total of 32 L of the respective solution over two consecutive days. At 25 days after pollination, three ears were randomly harvested from each plot (biological replicates). Total samples collected: 36 genotypes × 2 treatments × 3 replicates × 3 ears = 648 ears.

Harvested ears were thoroughly rinsed with deionized water, then oven-dried at 70 °C until constant weight was achieved. The kernels were manually removed from the cobs, ground using a laboratory mill, and passed through a 60-mesh sieve (250 μm pore size). The resulting powder was stored in airtight containers for subsequent determinations of: Micronutrient concentrations (Fe, Zn, Cu, Mn, Ca), Soluble sugar content, Crude protein content.

### 2.3 Measurement of agronomic traits

We collected five representative plants per plot at the milk stage (25 DAP) for agronomic trait evaluation. Plant height and ear height were measured using a tape measure (accuracy: 0.1 cm) from the base to the highest growing point and the top ear node, respectively; Fresh plant weight (roots excluded) was determined immediately after cutting using an electronic balance (accuracy: 0.1 g); Fresh ear weight (husks removed) was recorded immediately after harvest with the same balance; Ear length was measured as the absolute distance from the base (excluding peduncle) to the tip of the ear; Ear diameter was measured at the mid-section of the ear using a digital vernier caliper (accuracy: 0.01 mm). To account for ear irregularity, two perpendicular measurements were taken at each position, and the mean value was calculated.

### 2.4 Measurement of photosynthetic parameters

Photosynthetic parameters were determined on the 5th day after each CaCl_2_ foliar application under clear-sky conditions between 9:00–11:00 AM. Five uniformly growing plants per plot were randomly selected for leaf gas exchange measurements. The net photosynthetic rate (Pn) was determined using a portable photosynthesis system (CIRAS-3, PP Systems, USA). During V12 stage: the primary ear leaf. Measurements were conducted under controlled conditions. Reference CO_2_ concentration: 390 μL/L; Relative humidity: 60%; Photosynthetic photon flux density: 1600 μmol·m^-2^·s^-1^. Simultaneously, leaf chlorophyll content was quantified as SPAD values using a SPAD-502 chlorophyll meter (Konica Minolta, Japan) on the same leaves.

### 2.5 Determination of trace elements (Fe, Mn, Cu, Zn, and Ca)

A 0.5 g aliquot of oven-dried and finely ground waxy maize kernels was accurately weighed and digested with HNO_3_-H_2_O_2_ in a microwave accelerated reaction system (CEM, Matthews, NC, USA). The concentrations of Zn, Fe, Mn, Ca and Cu in the digested solutions were determined by inductively coupled plasma atomic emission spectroscopy (ICP-AES, OPTIMA 3300 DV, Perkin-Elmer, USA) (Xue et al., 2014).

### 2.6 Determination of protein content

A 0.5 g aliquot of oven-dried and finely ground waxy maize kernels was accurately weighed and digested with copper sulfate (CuSO_4_) and potassium sulfate (K_2_SO_4_) as catalysts. The total nitrogen (N) content was determined using the Kjeldahl method. The titration was carried out using a 0.05 mol/L hydrochloric acid (HCl) standard solution. Crude protein content was calculated by multiplying the total nitrogen content by a conversion factor of 6.25 (Gu et al., 2015).

### 2.7 Determination of soluble sugar content

A 0.1 g aliquot of oven-dried and finely ground waxy maize kernels was accurately weighed. Soluble sugars were extracted using 80% ethanol solution. The combined extracts were brought to a final volume of 10 mL. The reaction was carried out by incubating the extract with a freshly prepared anthrone-sulfuric acid reagent in a boiling water bath for 10 minutes. After cooling, the absorbance was measured at 620 nm. Glucose was used as the standard. The soluble sugar content was calculated according to the following formula: Soluble sugar content (mg/g) = (C × V × N)/(W × 1000), where C is the concentration determined from the standard curve (μg/mL), V is the final volume of the extract (mL), N is the dilution factor, and W is the dry weight of the sample (g) (Zhong et al., 2025).

### 2.8 Statistical analysis

All experiments were performed in triplicate. Data were presented as mean ± SE. All the tests were performed using SPSS Version 21.0 for Windows (SPSS, Chicago, IL, USA). Two-tailed Student’s *t*-test for independent samples, applied separately for each hybrid to compare control vs. CaCl_2_ treatment. Clearly defined as P < 0.05 (significant) and P < 0.01 (highly significant). Pearson’s correlation coefficient, with FDR (Benjamini–Hochberg) correction for multiple testing.

## 3 Results

### 3.1 Effects of foliar-applied calcium chloride on yield-related traits in waxy maize

To investigate the role of exogenous calcium in plant architecture and yield formation, we performed a foliar application experiment with 18 mmol/L CaCl_2_ on 36 waxy maize hybrids. With the exception of ‘Nongkeyu 336’, which showed a statistically significant 7.2% decrease, no other hybrids exhibited a significant reduction compared to the control. In contrast, no statistically significant effect was observed on ear height across the 36 evaluated waxy maize hybrids (**Table 1**). A significant decrease in fresh weight per plant was recorded for cultivars such as Runheinuo 829, Jingnuo 69, and Jingpo 82. Conversely, a significant increase was noted in hybrids including Runnuo 163, Runheinuo 629, Jingpo Zaonuo 18, Runnuo 615, and Runnuo 73. For fresh weight per ear, a trait directly linked to yield, three hybrids (Nongkeyu 336, Jinnuo 69, and Tianguinuo 932) showed a minor reduction. However, the majority (91.7%) of hybrids exhibited an increase, with 26 of them reaching statistical significance, demonstrating yield enhancements ranging from 2% to 21%. Notably, the CaCl_2_ treatment consistently and significantly improved yield-related morphological traits. Both ear length and ear diameter were positively affected (**Supplementary Figure 1**). Jinnuo 111 exhibited the largest increase in ear diameter (51.1%), while Jingkenuo 1 showed the most substantial improvement in ear length (33.2%). A significant increase in the number of kernels per row was observed in 30 waxy maize hybrids, with the maximum increase (42%) recorded in Wannuo 2000.

**Table 1:** Effects of calcium chloride (CaCl_2_) treatment on agronomic traits of 36 waxy maize hybrids.

Variety	CaCl_2_ concentration(mmol/L)	Plant height (cm)	Ear height (cm)	Fresh weight per plant (g)	Fresh weight per ear (g)
Jingkenuo 2000	0	120.3±2.1	227±16.8	1300±41.5	241.7±4.7
	18 mmol/L	112±4.6	223±9.6	1353.3±37.4	280±3.6**
Jingpo Bainuo	0	126±3.7	245.7±13.1	1343.3±46.8	277.8±5.5
	18 mmol/L	119±1.6	243.7±6.8	1366.9±28.9	292.1±8.4
Nongkeyu 336	0	120.7±2.5*	243.7±10.6	1076.1±53.3	304.4±8.8
	18 mmol/L	112±3.6	242±7.8	977.8±62.1	301.1±5.7
Jinnuo 215	0	127±5	256±9.9	1447.2±40.5	327.2±2.1
	18 mmol/L	125.3±4.5	261.3±13	1468.3±36	344.4±4.4**
Runheinuo 829	0	115±2.4	252±4.9	1235±41.4*	323.9±7.5
	18 mmol/L	115.7±4.9	253±4.5	1111.1±7.5	355±4.9**
Runnuo 72	0	107.7±2.9	219±7	836.7±18.4	287.2±9.6
	18 mmol/L	106.7±3.3	220.7±2.6	831.7±31.3	308.9±4.2*
Runnuo 163	0	108±5.7	219±11.5	807.2±15.2	179.4±6.7
	18 mmol/L	106±5.7	221.7±7.4	921.7±32.5**	203.3±7.2*
Runcainuo 686	0	117.3±3.1	256±5.9	1222.2±44.4	320.3±6.2
	18 mmol/L	114.7±1.7	250±6.2	1226.7±51.7	347.2±5.2**
Jinnuo 69	0	103.7±1.7	199±5	818.9±24.5**	306.7±5.9
	18 mmol/L	97.3±3.9	190.7±4.6	725±13.4	303.3±5.9
Runnuo 1704	0	105.7±5.2	231±5.7	851.1±40.9	260.6±6.7
	18 mmol/L	101.7±3.9	225.3±4.2	870.8±32.3	298.9±3.9**
Wannuo 2000	0	136±4.3	259.7±1.7	1186.1±51.7	261.7±9.5
	18 mmol/L	134.3±5.2	257.3±4.9	1207.8±43.2	303.8±8.9**
Jingkenuo 1	0	113±3.3	256.3±7.3	976.1±54.3	258.3±9.8
	18 mmol/L	116±4.5	255.3±8.2	911.8±21.4	290.8±5.6**
Runheinuo 729	0	115.3±4.6	240.7±8.4	1085±65.9*	303.9±6.7
	18 mmol/L	116±3.7	244.7±4.5	848.9±63.3	306.1±5.2
Jingpo 82	0	109±4.2	236.7±7.3	1091.1±57.8	308.3±7.6
	18 mmol/L	113.7±4.1	249.3±10.9	1114.1±24.5	336.1±9.1*
Runheinuo 529	0	115.3±3.9	230.3±7.7	878.9±23.9	289.4±7.5
	18 mmol/L	110.7±4.6	235.3±4.2	911.1±25	302.8±5.2
Jinnuo 72	0	122.7±4.1	240±8.3	1371.1±76.5	273.9±6.8
	18 mmol/L	117±2.9	243.7±7.7	1428.9±56.4	313.4±7.7**
Jingpo Yinnuo	0	122±4.6	259.3±8.2	1288.9±37.8	306.1±4.2
	18 mmol/L	117.3±1.2	267±4.9	1371.7±21.3*	339±5.6**
Runheinuo 629	0	114.3±4.5	250.7±6.6	1172.2±56.5	222.2±17
	18 mmol/L	113.7±3.4	245±5.9	1257.2±61.5	263.8±4.1*
Runnuo 622	0	120.7±5.6	246.7±5.2	1213.3±70.9	295.6±9.3
	18 mmol/L	116±4.3	249±9.8	1287.8±68.3	319.4±5.5*
Runnuo 175	0	108.3±1.3	215.3±6	1291.1±61.7	324.4±4.4
	18 mmol/L	111.7±1.2	218±6.4	1237.2±14.4	348.9±3.1**
Jingnuo 307	0	97.3±3.7	201±7	831.1±72.9	317.8±3.4
	18 mmol/L	98.3±1.2	201.7±4.2	713.3±46.8	324.4±5.2*
Runnuo 605	0	117.7±1.7	242.7±4.1	1176.7±103.6	308.9±8
	18 mmol/L	112.3±2.5	238.3±11.1	1167.2±30.2	341.7±9.5*
Jingpo Zao 18	0	120±4.3	237±3.3	887.8±9.6	252.8±4.8
	18 mmol/L	116±2.9	241.3±3.7	940±9.8**	295.8±3.6**
Jingpo 556	0	109±1.6	223.3±2.1	803.3±74.9	306.1±5.5
	18 mmol/L	105.3±5.4	220.3±9	828.9±25.5	336.7±10.8*
Runnuo 597	0	104±3.6	225±5.9	1598.3±79.6	316.1±2.8
	18 mmol/L	108.3±3.9	216.3±9.5	1311.7±64.8	319.8±3
Runnuo 615	0	112.3±3.9	234.7±4.9	1035.6±55.1	263.3±3.6
	18 mmol/L	109.3±4.1	238.7±8.6	1199.2±43.7*	305.2±10.8**
Jingpo Zaonuo	0	105±3.3	221.3±7.8	912.4±15.9	271.1±8.7
	18 mmol/L	113.3±3.3	218±3.6	951.6±32.4	303.7±9.4*
Tianguinuo 932	0	128.7±6.3	250.3±2.9	1165.6±72.9	324.4±5.2
	18 mmol/L	127.3±2.9	264.7±7	1167.8±24	318.9±7.9
Runnuo 73	0	108.7±2.5	213.3±13.2	826±10.4	241.1±4.2
	18 mmol/L	103.3±3.3	214.7±7	870±10.3*	292.6±6.9**
Lixiang Hongnuo	0	108±6.7	215±4.3	830±33.4	252.2±9.6
	18 mmol/L	113.7±4.1	223±4.9	852±19.5	288.4±7.4*
Jinnuo 111	0	120.34±4.8	237±3.3	1542.9±48.2	342.2±2.8
	18 mmol/L	117.3±3.9	240.7±2.1	1406.7±63.6	379.4±4.2**
Runnuo 863	0	115.3±5.3	203.7±9.1	1112.2±29.3	236.1±10.3
	18 mmol/L	113±5.7	200.7±13.2	1185±51	248.9±2.1
Huangnuo Zao 53	0	131.7±3.9	255.7±9.8	1498.9±78.7	315±9.5
	18 mmol/L	129.7±2.1	243.7±6.8	1414.4±85.7	317.2±5.7
Runnuo 197	0	111±4.3	241.3±8.5	1323.3±59.2	279.4±8
	18 mmol/L	111.3±4	235.3±6.2	1295.6±40.6	303.1±4.5*
Runnuo 650	0	121.3±4.8	231.7±4.1	1167.8±82.8	290.6±10.3
	18 mmol/L	119.3±5.8	238.7±4.1	1096.7±43	295±11.8
Dahuangnian	0	142.7±4.5	251.7±5.7	1135.6±88	145±3.6
	18 mmol/L	136.3±4.5	248±6.2	1216.1±52.4	160±3.6*

Data are means ± standard deviation (n = 3 replications of five plants). Asterisks denote a significant difference according to an unpaired Student’s t-test (*: p < 0.05; **: p < 0.01). The same applies below.

### 3.2 Foliar application of calcium chloride enhances photosynthesis in waxy maize

**Figure 1:**
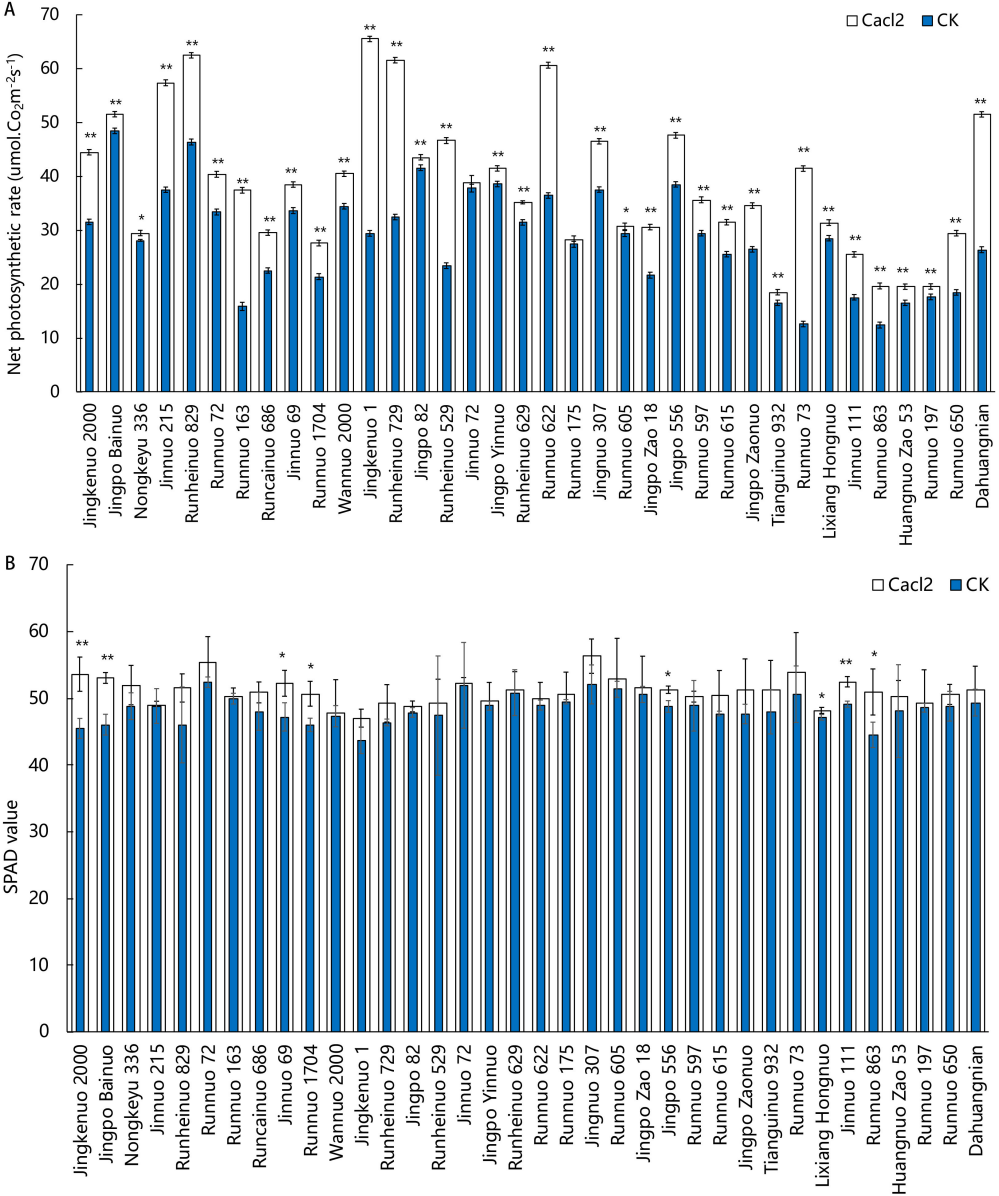
Effects of calcium chloride treatment on SPAD value and net photosynthetic rate at the tasseling stage. **(A)** Net Photosynthetic Rate. **(B)** Relative chlorophyll content (SPAD value). Data are presented as mean ± SEM (n = 3). Asterisks indicate significant differences between control and CaCl_2_ treatment within the same hybrid: *P < 0.05, P < 0.01 (Student’s t-test).

To assess the effects of a foliar application of calcium on photosynthesis, we measured Pn and SPAD values for the leaves of waxy maize hybrids treated with exogenous CaCl2. A pronounced physiological response was observed at the bell stage. Pn values increased significantly for 34 of the 36 analyzed waxy maize hybrids (exceptions were ‘Jinnuo 72’ and ‘Runnuo 175’), with increases ranging from 4.5% to 230% (‘Runnuo 73’) (**Figure 1A**). Furthermore, SPAD values for ear leaves showed an increasing trend across all hybrids. Significant increases (P < 0.05) were detected in eight hybrids, with increments ranging from 2.2% to 17.9% (**Figure 1B**). Among these, ‘Jingkenuo 2000’ exhibited the largest increase (17.9%), followed by ‘Jingpo Bainuo’ (15.2%) and ‘Runnuo 863’ (14.4%) (**Figure 1B**).

### 3.3 Foliar calcium chloride selectively enhances iron, zinc, and calcium accumulation in waxy maize kernels

To assess the potential of calcium treatment for kernels nutritional fortification, we analyzed the micronutrient content in kernels following foliar CaCl_2_ application. Exogenous CaCl_2_ application significantly influenced the accumulation of several micronutrients in the kernels of waxy maize, although the effect strength demonstrated distinct element specificity (**Table 2**). The CaCl_2_ treatment generally promoted the accumulation of iron (Fe), zinc (Zn), and calcium (Ca) in the kernels. Among the 36 hybrids, 33 showed a significant increase in Fe content following CaCl_2_ treatment. The remaining three hybrids (‘Jingpo Bainuo’, ‘Jingpo Zaonuo’, and ‘Lixiang Hongnuo’) exhibited no significant change (P > 0.05). A significant increase in Zn content was detected in 31 hybrids. Five hybrids (‘Jingpo Bainuo’, ‘Runnuo 175’, ‘Jingnuo 307’, ‘Jingpo Zaonuo’, and ‘Lixiang Hongnuo’) showed no significant response. Significant enhancement of Ca content was observed in 32 hybrids. The remaining four hybrids (‘Jingpo Bainuo’, ‘Jingnuo 307’, ‘Jingpo Zao 18’, and ‘Jingpo Zaonuo’) did not show a significant increase. Notably, 30 hybrids exhibited a simultaneous and significant increase in the contents of all three aforementioned micronutrients compared to the control. In contrast, the response of copper (Cu) and manganese (Mn) was more limited. Significant increases in their content were detected in 11 hybrids (31%), though all measured values for these micronutrients showed an increasing trend relative to the control group.

**Table 2:** Effects of calcium chloride (CaCl_2_) treatment on micronutrient content in Kernels of 36 waxy maize hybrids.

Variety	CaC_2_ concentration (mmol/L)	Iron content (mg/kg)	Copper content (mg/kg)	Zinc content (mg/kg)	Manganese content (mg/kg)	Calcium content (mg/kg)
Jingkenuo 2000	0	36.3±0.26	1.7±0.1	15.33±0.25	4.23±0.12	29.53±0.12
	18 mmol/L	38.5±0.26**	1.83±0.06	16.53±0.12**	4.37±0.06	31.33±0.15**
Jingpo Bainuo	0	34.2±0.1	1.37±0.06	12.23±0.15	3.57±0.23	25.5±0.17
	18 mmol/L	34.37±0.25	1.43±0.06	12.27±0.06	3.63±0.12	25.63±0.06
Nongkeyu 336	0	37.3±0.26	1.6±0.17	14.23±0.21	3.77±0.23	33.43±0.21
	18 mmol/L	38.8±0.26**	1.63±0.06	14.63±0.06*	3.8±0.1	34.17±0.06**
Jinnuo 215	0	38.43±0.25	1.53±0.12	14.53±0.21	3.53±0.12	34.53±0.12
	18 mmol/L	40.37±0.21**	1.67±0.06	15.47±0.21**	3.73±0.06	35.97±0.12**
Runheinuo 829	0	43.33±0.15	1.67±0.15	13.07±0.15	3.33±0.12	33.7±0.17
	18 mmol/L	46.87±0.25**	1.97±0.06*	14.13±0.06**	3.53±0.06	36.63±0.12**
Runnuo 72	0	40.33±0.32	1.2±0.17	14.47±0.23	3.63±0.29	35.4±0.17
	18 mmol/L	44.67±0.32**	1.33±0.06	15.6±0.17**	3.8±0.1	36.87±0.06**
Runnuo 163	0	45.37±0.21	1.4±0.17	14.7±0.17	3.37±0.23	35.6±0.17
	18 mmol/L	51.33±0.25**	1.83±0.06*	16.5±0.26**	4.17±0.06*	39.23±0.15**
Runcainuo 686	0	35.23±0.12	1.07±0.15	10.9±0.2	3.3±0.17	33.37±0.23
	18 mmol/L	38.3±0.26**	1.27±0.06	12.17±0.06**	3.6±0.1	36.53±0.23**
Jinnuo 69	0	39.43±0.21	1.23±0.12	13.33±0.21	3.53±0.12	32.7±0.17
	18 mmol/L	43.67±0.21**	1.37±0.06	14.6±0.17**	3.77±0.06*	34.3±0.17**
Runnuo 1704	0	41.27±0.21	1.17±0.06	12.13±0.15	3.3±0.17	33.33±0.12
	18 mmol/L	44.67±0.21**	1.27±0.06	12.77±0.15**	3.53±0.06	36.2±0.17**
Wannuo 2000	0	35.63±0.15	1.1±0.1	12.17±0.12	3.37±0.23	33.5±0.17
	18 mmol/L	38.93±0.15**	1.37±0.06*	13.47±0.12**	3.73±0.06	36.6±0.17**
Jingkenuo 1	0	34.43±0.32	1.03±0.12	10.07±0.15	3.17±0.06	25.47±0.23
	18 mmol/L	36.17±0.31**	1.07±0.06	10.37±0.06*	3.4±0.1*	26.97±0.12**
Runheinuo 729	0	36.33±0.4	1.17±0.06	11.83±0.12	3.37±0.23	26.13±0.12
	18 mmol/L	40.33±0.25**	1.5±0.1*	13.3±0.1**	3.83±0.06*	29.27±0.06**
Jingpo 82	0	34.57±0.15	1.23±0.06	13.4±0.2	3.37±0.06	24.43±0.12
	18 mmol/L	36.87±0.21**	1.33±0.06	13.83±0.06*	3.47±0.06	25.3±0.26**
Runheinuo 529	0	43.37±0.21	1.33±0.06	13.47±0.23	3.23±0.12	26.6±0.17
	18 mmol/L	47.73±0.32**	1.47±0.06*	15.27±0.15**	3.57±0.06*	29.27±0.15**
Jinnuo 72	0	35.37±0.21	1.17±0.12	12±0.1	3.57±0.23	23.27±0.15
	18 mmol/L	36.6±0.36**	1.27±0.12	13.23±0.06**	3.77±0.15	26.37±0.12**
Jingpo Yinnuo	0	34.63±0.25	1.2±0.1	11.17±0.15	3.2±0.1	22.13±0.21
	18 mmol/L	35.8±0.26**	1.27±0.06	11.6±0.1*	3.33±0.06	23.27±0.15**
Runheinuo 629	0	39.07±0.15	1.2±0.1	13.1±0.17	3.23±0.12	25.23±0.12
	18 mmol/L	45.07±0.25**	1.47±0.06*	14.37±0.15**	3.57±0.12*	28.0±0.17**
Runnuo 622	0	42.5±0.26	1.23±0.12	12.33±0.12	3.47±0.23	24.23±0.12
	18 mmol/L	48.97±0.74**	1.67±0.06**	14.03±0.12**	4.13±0.06**	27.93±0.15**
Runnuo 175	0	41.33±0.25	1.27±0.15	14.87±0.06	3.53±0.12	26.73±0.12
	18 mmol/L	44.57±0.31**	1.3±0	15.03±0.12	3.67±0.06	27.73±0.21**
Jingnuo 307	0	38.43±0.15	1.17±0.12	12.27±0.15	3.23±0.12	22.87±0.4
	18 mmol/L	39.37±0.25**	1.23±0.06	12.37±0.06	3.33±0.06	23.1±0.46
Runnuo 605	0	39.6±0.26	1.57±0.06	14.5±0.17	3.33±0.12	28.53±0.12
	18 mmol/L	42.87±0.21**	1.67±0.06	15.63±0.06**	3.73±0.06*	30.27±0.06**
Jingpo Zao 18	0	36.73±0.35	1.47±0.06	13.63±0.12	3.27±0.15	22.3±0.17
	18 mmol/L	39±0.36**	1.53±0.06	14.17±0.06**	3.33±0.06	22.63±0.21
Jingpo 556	0	37.5±0.3	1.17±0.06	14.03±0.12	3.5±0.17	23.67±0.23
	18 mmol/L	40.07±0.25**	1.27±0.06	15.23±0.12**	3.7±0.1	24.17±0.12*
Runnuo 597	0	41.27±0.21	1.27±0.15	12.93±0.29	3.67±0.23	25.5±0.17
	18 mmol/L	46.83±0.15**	1.7±0.1*	14.63±0.15**	4.27±0.06*	28.63±0.21**
Runnuo 615	0	43.37±0.25	1.87±0.12	13.23±0.12	3.77±0.23	35.53±0.12
	18 mmol/L	48.6±0.26**	2.2±0.1*	14.5±0.17**	4.23±0.06*	38.57±0.15**
Jingpo Zaonuo	0	36.23±0.15	1.8±0.17	14.4±0.26	3.23±0.12	28.33±0.12
	18 mmol/L	36.7±0.26	1.87±0.06	14.5±0.17	3.3±0.1	28.53±0.21
Tianguinuo 932	0	38.37±0.15	1.57±0.12	11.23±0.12	3.17±0.06	29.53±0.12
	18 mmol/L	39±0.17**	1.57±0.06	11.53±0.06*	3.2±0	30.0±0.17*
Runnuo 73	0	41.37±0.21	1.2±0.1	13.23±0.15	3.37±0.23	34.23±0.12
	18 mmol/L	45.03±0.21**	1.33±0.06	13.77±0.15*	3.63±0.06	36.27±0.21**
Lixiang Hongnuo	0	39.57±0.15	1.17±0.15	12.13±0.06	3.3±0.17	28.53±0.12
	18 mmol/L	39.93±0.29	1.2±0	12.23±0.06	3.4±0.1	28.77±0.06*
Jinnuo 111	0	37±0.1	1.33±0.06	11.23±0.12	3.53±0.12	28.47±0.23
	18 mmol/L	39.4±0.26**	1.43±0.06	12.2±0.17**	3.67±0.06	31.3±0.17**
Runnuo 863	0	39.67±0.38	2.17±0.06	15.17±0.06	3.77±0.15	34.3±0.17
	18 mmol/L	43.5±0.26**	2.27±0.06	16.43±0.15**	4.03±0.12	36.37±0.21**
Huangnuo Zao 53	0	36.93±0.15	1.47±0.06	12.27±0.15	3.3±0.17	31.03±0.12
	18 mmol/L	38.3±0.26**	1.63±0.06*	13.17±0.06**	3.47±0.12	32.17±0.12**
Runnuo 197	0	37.27±0.21	1.27±0.12	12.33±0.12	3.47±0.23	28.8±0.17
	18 mmol/L	39.73±0.21**	1.3±0	12.83±0.12*	3.77±0.06	32.23±0.12**
Runnuo 650	0	38.67±0.21	1.77±0.15	14.5±0.17	3.63±0.12	30.03±0.12
	18 mmol/L	41.5±0.26**	1.87±0.15	15.0±0.17*	3.87±0.06*	33.3±0.26**
Dahuangnian	0	43.47±0.25	1.77±0.15	14.17±0.12	3.27±0.15	29.33±0.15
	18 mmol/L	46.53±0.21**	2.13±0.06*	15.27±0.15**	3.43±0.06	31.37±0.21**

Data are means ± standard deviation (n = 3 replications of five plants). Asterisks denote a significant difference according to an unpaired Student’s t-test (*: p < 0.05; **: p < 0.01).

### 3.4 Effect of calcium chloride treatment on soluble sugar and crude protein content in waxy maize kernels

**Figure 2:**
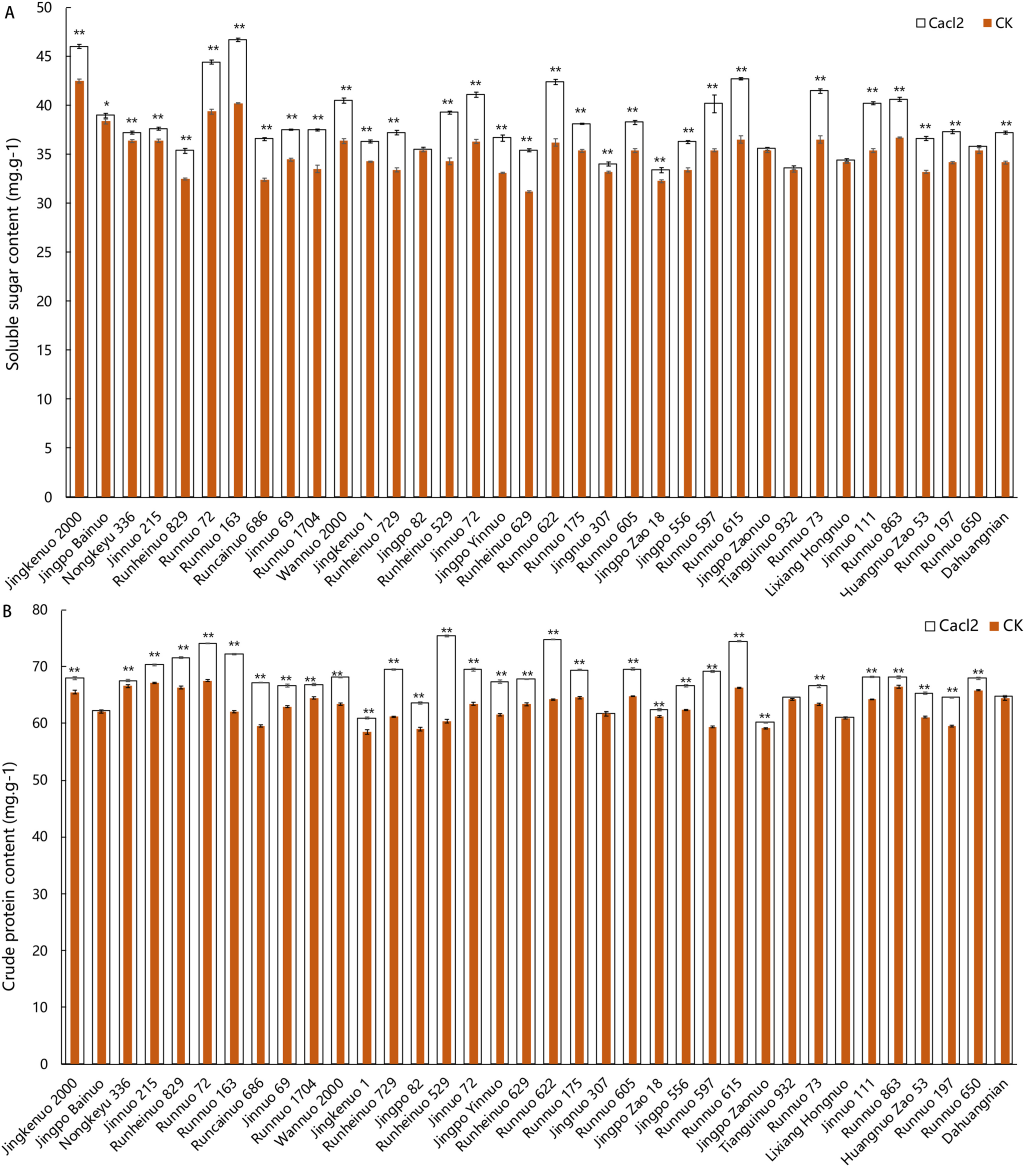
Effects of calcium chloride treatment on soluble sugar and crude protein content in kernels of 36 waxy maize hybrids. **(A)** Soluble Sugar content; **(B)** Crude Protein Content. Data are presented as mean ± SEM (n = 3). Asterisks indicate significant differences between control and CaCl_2_ treatment within the same hybrid: *P < 0.05, **P < 0.01 (Student’s t-test).

The CaCl_2_ treatment significantly influenced the nutritional quality of waxy maize kernels. Regarding soluble sugar content, 31 out of the 36 hybrids (86.1%) exhibited a significant increase compared to the control, with the exceptions being Jingpo 82, Jingpo Zaonuo, Lixiang Hongnuo, Tiangui Nuo 932, and Runnuo 650 (**Figure 2A**). A similar widespread increasing trend was observed for crude protein content. With the exception of Jingpo Zaonuo, Tiangui Nuo 932, Lixiang Hongnuo, and Dahuang Nian, the crude protein accumulation in the remaining varieties showed a significant difference from the control group (**Figure 2B**).

**Figure 3:**
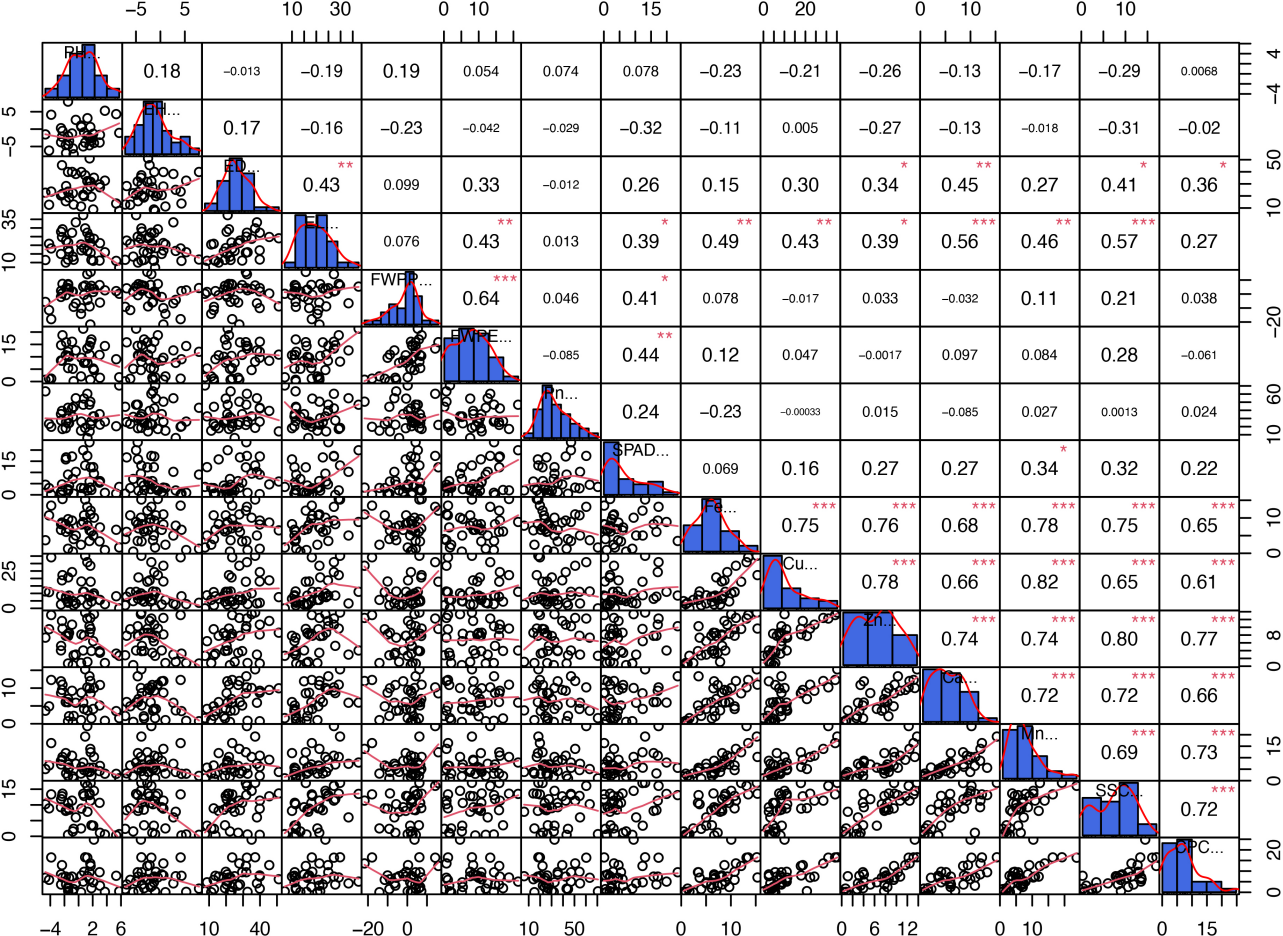
Correlation analysis of the measured indicators. PH, Plant height; EH, Ear height; ED, Ear diameter; EL, Ear length; FWPP, Fresh weight per plant; FWPE, Fresh weight per ear; Pn, Net Photosynthetic Rate; Fe, Iron; Cu, Copper; Zn, Zinc; Ca, Calcium; Mg, Manganese; SSC, Soluble Sugar content; CPC, Crude Protein Conten. *P < 0.05; **P < 0.01; ***P < 0.001.

To further elucidate the differences in response patterns among varieties, a quadrant analysis was performed based on the change rates of soluble sugar and crude protein (**Supplementary Figure 2**). The results indicated that the 36 hybrids could be classified into four distinct nutritional quality response types: i) High-Sugar-High-Protein: This group, including nine varieties such as Runnuo 163, showed a synergistic and significant increase in both soluble sugar and crude protein content after treatment. ii) Sugar-Specific: Represented by five varieties, including Jinnuo 111, this type exhibited a particularly sensitive response in sugar accumulation to the calcium treatment, while protein content remained unchanged. iii) Protein-Specific: Only one variety, Jingpo 82, belonged to this category, demonstrating a specific protein-fortifying capability.

### 3.5 Multi-trait association analysis

A correlation analysis was performed on the variation rates of major agronomic traits, micronutrient content, crude protein content, and soluble sugar content in the kernels of the 36 waxy maize hybrids. The results indicated that the variation rate of soluble sugar content in the kernels was significantly correlated with the variation rates of ear length and ear diameter. Furthermore, significant positive correlations were identified between the variation rates of micronutrients (Fe, Mg, Zn, Cu, Ca) and the variation rates of both crude protein and soluble sugar content (**Figure 3**). Collectively, these correlations reveal the intrinsic linkages between sink strength (ear development), carbon allocation (sugar), and nutritional metabolism, providing a physiological basis for the synergistic yield and quality improvement induced by calcium.

## 4 Discussion

This study demonstrates that exogenous CaCl_2_ treatment, despite causing a slight suppression of plant and ear height, significantly enhanced ear traits—manifested as increased ear length, ear diameter and Kerneles per row (**Supplementary Figure 1**) and a widespread improvement in fresh weight per ear, 72% of the hybrids (26 varieties) showed a significant increase compared to the control (**Table 1**). This seemingly paradoxical phenotype reveals that the core role of calcium signaling lies in reprogramming the partitioning of photoassimilates, rather than simply inhibiting or promoting growth. This inhibiting vegetative growth while promoting reproductive growth effect may originate from dual pathways involving calcium-mediated morphogenesis and hormonal regulation. On one hand, as a key bridging ion for structural components of the cell wall (e.g., calcium pectate), calcium contributes to enhanced cell wall rigidity and stability (Hepler, 2005). Tissue softening is delayed through enhanced cell wall stability (Guo et al., 2023). It is plausible that calcium reinforces the mechanical strength of the cell wall, thereby physically constraining excessive cell elongation and limiting plant height. On the other hand, functioning as a crucial second messenger, calcium signaling is deeply involved in modulating the endogenous hormone network. It may activate the synthesis or signaling transduction of cytokinins (Anil et al., 2000; Frattini et al., 1999; Morello et al., 2000), thereby coordinating the development of female florets to increase kernel number per ear and enhancing the kernel filling intensity, ultimately strengthening the sink capacity of the reproductive organs.

This study observed that CaCl_2_ treatment significantly enhanced the net photosynthetic rate of waxy maize without markedly altering the relative chlorophyll content (SPAD value). This phenomenon indicates that the promotion of photosynthesis by exogenous calcium primarily stems from an optimization of the operational efficiency of the photosynthetic apparatus, rather than an expansion of its light-harvesting capacity. This decoupling effect resembles the performance associated with reported functional stay-green traits (Hörtensteiner, 2009). As a pivotal second messenger, the calcium signaling network responds to various environmental and hormonal signals, enabling the precise regulation of ion channels in guard cells and thereby optimizing stomatal aperture (McAinsh and Pittman, 2009; Yang et al., 2022; Zhang et al., 2020). In this study, calcium treatment may have provided leaves with a more ample intercellular CO_2_ concentration without substantially increasing transpirational costs, thereby directly driving the enhancement of carbon fixation.

At the chloroplast level, the stabilization of thylakoid membranes by calcium ions optimizes the photosynthetic electron transport chain. Research indicates that exogenous calcium safeguards PSII reaction centers and maintains high electron transfer efficiency from QA to QB (Huang et al., 2019), thus securing efficient light-to-chemical energy conversion and providing ample ATP and NADPH for the dark reactions. At the carbon assimilation stage, calcium signaling is further involved in regulating the activation of Rubisco, the key enzyme in the Calvin cycle. Studies in honeysuckle have revealed that exogenous calcium can induce overexpression of the *rbcL* gene (encoding the large subunit of Rubisco) under stress conditions (Huang et al., 2019), Concurrently, calcium-dependent protein kinases may indirectly maintain its efficient carboxylation function by modulating Rubisco activase activity (Sharma and Komatsu, 2002). It is precisely this multi-level, systemic orchestration of the photosynthetic hardware from stomatal behavior, light energy capture, and electron transport to carbon fixation by calcium signaling that comprehensively enhances the overall efficiency of energy and matter conversion without increasing chlorophyll content, ultimately leading to a significant increase in the net photosynthetic rate.

The significant increase in essential micronutrients such as zinc (Zn) and iron (Fe) in the kernels highlights the considerable potential of calcium in mineral biofortification. This phenomenon may originate from the precise regulatory control of calcium signaling over the entire sequence of mineral processes, from uptake to translocation (Wang et al., 2023). Specifically, calcium signaling, potentially through pathways such as CBL(calcineurin B-like proteins)-interacting protein kinases, can modulate the expression of root transporters (e.g., IRT1, ZIP families), thereby synchronously enhancing the entire transport pathway of micronutrients like Zn and Fe from the rhizosphere to the kernels (Ju et al., 2022; Tang et al., 2023; Zhang et al., 2023). The concurrent increase in soluble sugar and crude protein content under calcium treatment can be attributed to a combination of factors. The elevated photosynthetic rate provides abundant carbon skeletons (in the form of soluble sugars), while calcium signaling may directly activate nitrogen metabolism. These two effects collectively supply sufficient substrates and driving force for protein synthesis. Consequently, the calcium treatment achieves a comprehensive enhancement of kernel nutritional quality.

**Figure 4:**
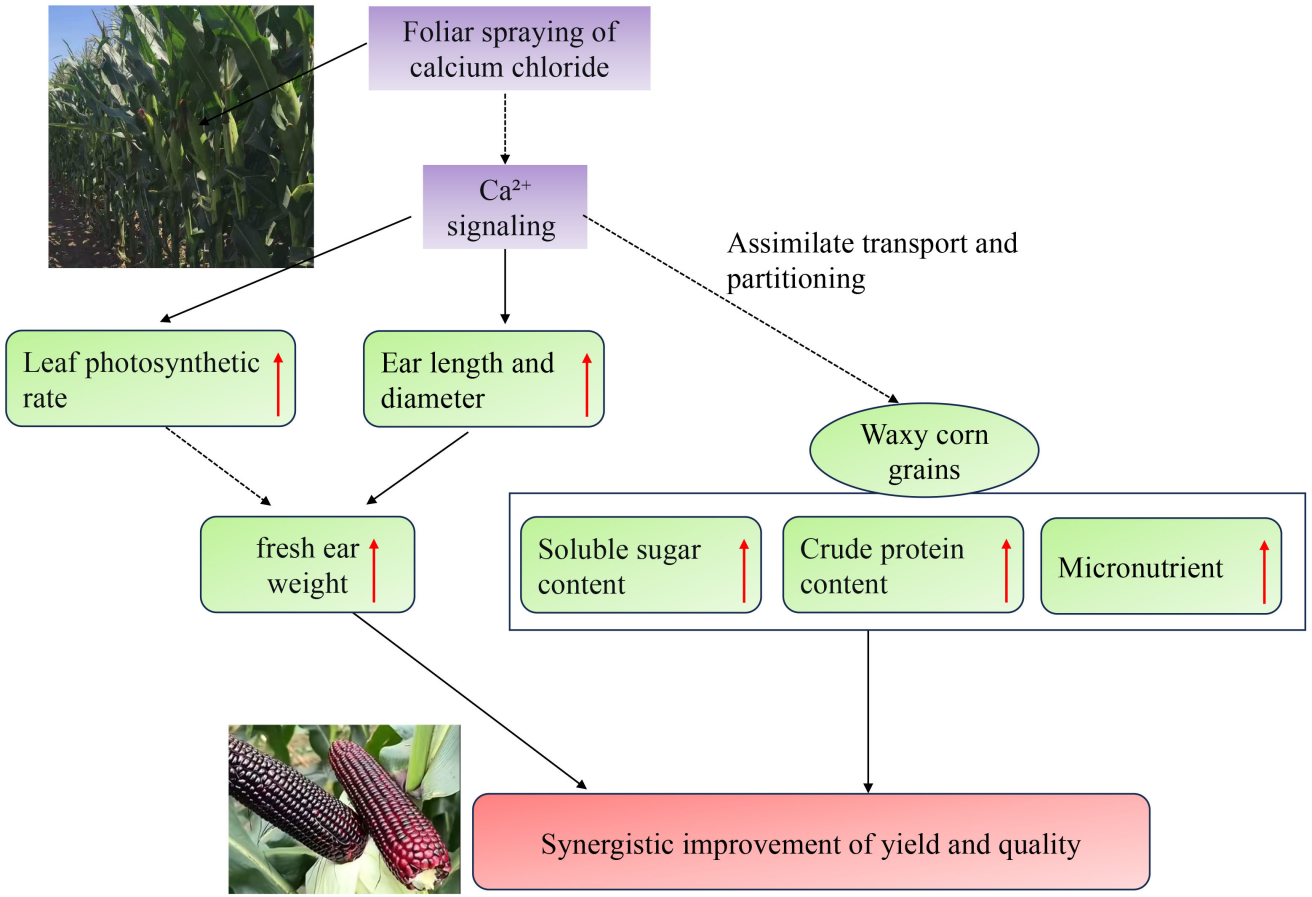
A possible network underlying the synergistic enhancement of yield and quality in response to calcium chloride treatment. Red arrows indicate an increase in the measured parameters.

Based on the aforementioned experimental results, we propose a novel, integrated physiological model that provides a mechanistic framework for the synergistic enhancement of yield and nutritional quality in waxy maize following foliar calcium treatment (**Figure 4**). This model posits that exogenous calcium acts as a systemic signal orchestrator rather than a mere nutrient, initiating a coordinated reprogramming of plant physiology. The central tenet of this model is the calcium-mediated optimization of the source-sink relationship. Upon application, calcium signaling is activated, which concurrently enhances the photosynthetic capacity (source strength) by improving the operational efficiency of the photosynthetic apparatus (as evidenced by increased Pn without higher SPAD), and strengthens the sink organs by promoting ear development (increased length and diameter). This dual enhancement creates a more efficient assimilate flux, directly driving the increase in fresh weight per ear—the core yield component. Critically, the model extends beyond yield architecture to explain integrated nutrient partitioning. We hypothesize that the same calcium signal facilitates not only the preferential flow of photoassimilates (e.g., sugars) but also orchestrates the coordinated root-to-kernel transport of minerals. This ensures that the enhanced carbon skeletons and the activated nitrogen metabolism are complemented by a sufficient supply of micronutrients (Fe, Zn, Ca, etc.), leading to the simultaneous enrichment of kernels with soluble sugars, crude protein, and essential minerals.

It elevates foliar calcium application from a simple agronomic practice to a strategic tool for synergistic biofortification, simultaneously addressing yield and multiple nutritional gaps (protein, sugars, and key micronutrients). However, future studies involving multi-year, multi-location trials across representative maize-growing regions are necessary to validate the stability of these responses and to develop environment-specific calcium application strategies.

## 5 Conclusion

The application of exogenous CaCl_2_ improved yield-related traits (ear length, diameter, and fresh weight) while reducing plant and ear height. It enhanced carbon assimilation by optimizing photosynthetic efficiency instead of increasing chlorophyll content. Furthermore, it concurrently enriched the kernels with organic components (soluble sugars, protein) and micronutrients (Zn, Fe, Ca), effectively coordinating the simultaneous improvement of yield and nutritional quality. Our study provides both a practical agronomic tool and a physiological framework for the integrated improvement of cereal crops.

## Data Availability

The original contributions presented in the study are included in the article/**Supplementary Material**. Further inquiries can be directed to the corresponding author/s.
