# Supplementary material for: Calcium application synergistically enhances yield and nutritional quality in waxy maize
Source: Front Plant Sci. 2026 Mar 11;17:1777765. doi: 10.3389/fpls.2026.1777765 (PMC13013329; doi:10.3389/fpls.2026.1777765)
Supplement: Supplementary file 1 [file Supplementaryfile1.docx]

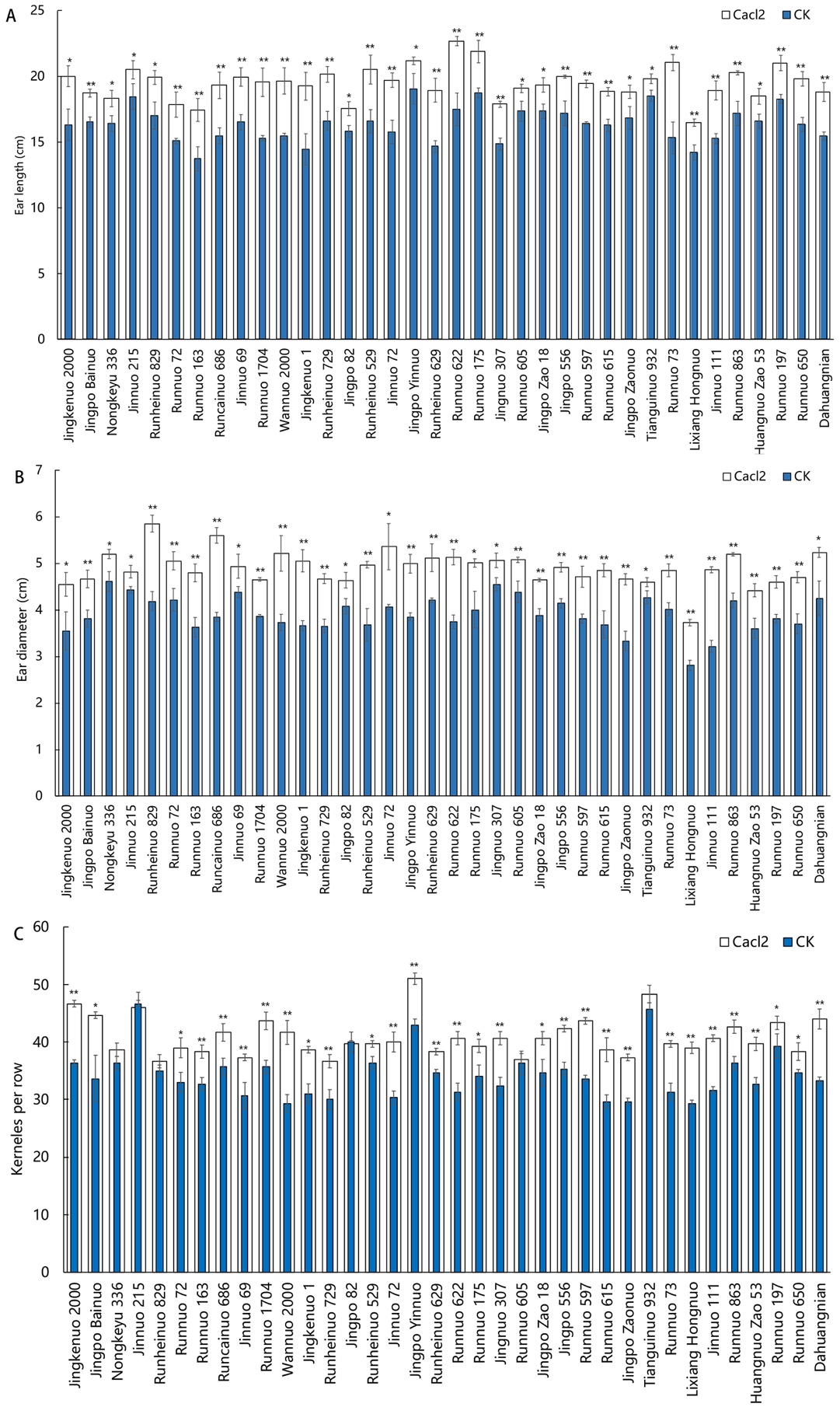


Supplementary Fig. 1. Effect of Calcium Chloride Treatment on Ear Length, Diameter and Kerneles per row.

(A) Ear length, (B) Ear diameter, (C) Kerneles per row. Data are presented as mean ± SEM (n = 3). Asterisks indicate significant differences between control and CaCl₂ treatment within the same hybrid: *P < 0.05, P < 0.01 (Student's t-test).


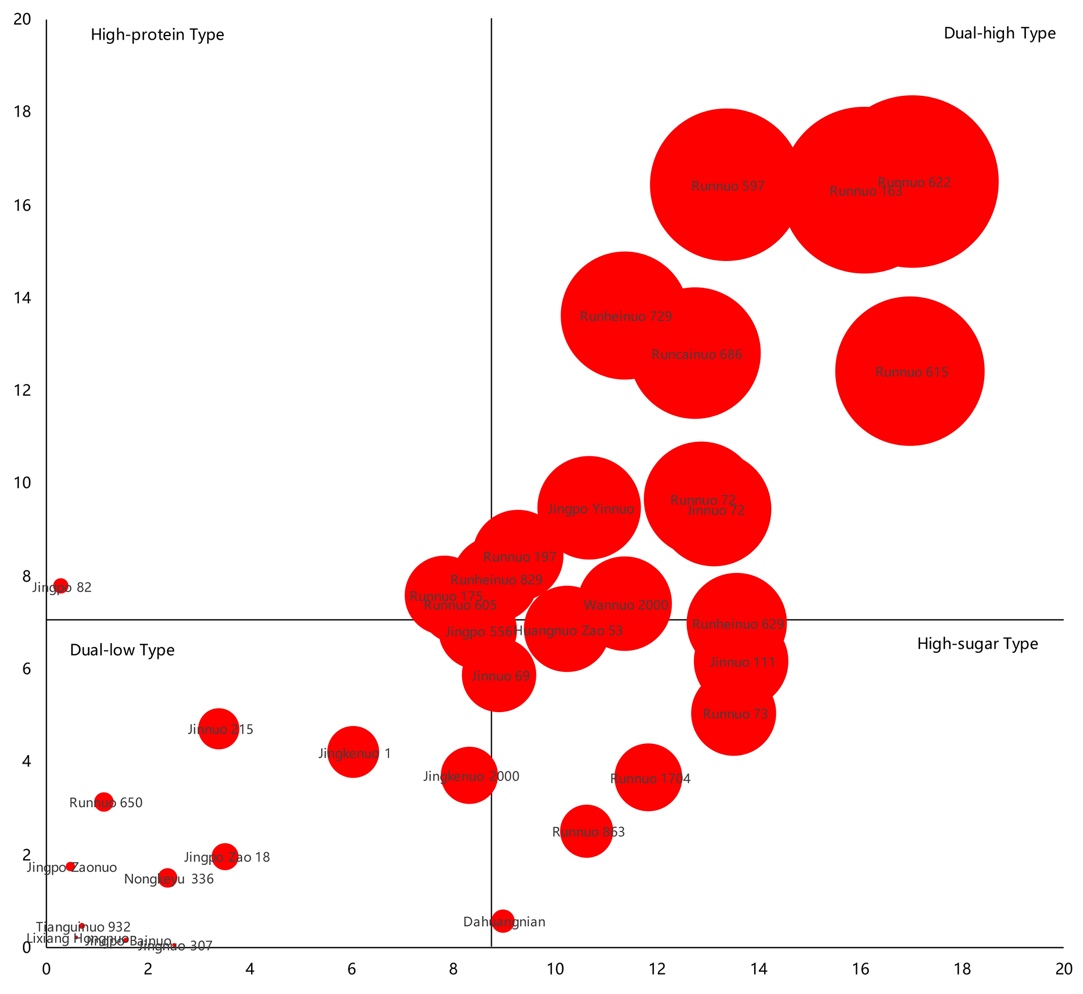


Supplementary Fig. 2. Quadrant Analysis of Variation Rates in Soluble Sugar and Crude Protein Content
